# High‐Yield Expression of Arylmalonate Decarboxylase in *Escherichia coli* Through High‐Cell‐Density Cultivation Strategies

**DOI:** 10.1002/cbic.70339

**Published:** 2026-04-21

**Authors:** Jan Gerstenberger, Timm Werbilo, Robert Kourist, Selin Kara

**Affiliations:** ^1^ Institute of Technical Chemistry Leibniz University Hannover Hannover Germany; ^2^ Institute of Molecular Biotechnology Graz University of Technology Graz Austria; ^3^ Biocatalysis and Bioprocessing Group Department of Biological and Chemical Engineering Aarhus University Aarhus C Denmark

**Keywords:** arylmalonate decarboxylase, biocatalysis, *E. coli* BL21 (DE3), enzyme expression, high‐cell‐density cultivation

## Abstract

This study develops and optimizes a high‐cell‐density cultivation (HCDC) of *E. coli* BL21 (DE3) to improve enzyme production utilizing DASGIP multibioreactor systems. By shifting from traditional shake flask methods to HCDC, we observed a 43‐fold increase in biomass production, with a comparable mass‐specific activity of the cell‐free extract. The HCDC demonstrated a 30‐fold increase in the active, soluble expression of arylmalonate decarboxylase (AMDase) from *Bordetella bronchiseptica* (*Bb*AMDase) compared to conventional methodology. The protocol was designed to be readily adapted to various expression approaches and to provide a robust foundation for broader use in recombinant protein production. The HCDC was achieved with a linear glucose feed to promote robust cell growth. We optimized the glycerol feeding strategy during isopropyl *β*‐D‐1‐thiogalactopyranoside (IPTG) induction to maximize AMDase production. Throughout the induction phase, we monitored both soluble expression yield and enzymatic activity, aiming to establish a process that is not only profitable but also efficient and stable.

## Graphical abstract

Optimized high‐cell‐density cultivation in the DASGIP® multibioreactor enabled a 30‐fold increase in soluble expression of arylmalonate decarboxylase (*Bb*AMDase) from *Bordetella bronchiseptica* in *E. coli* BL21 (DE3). Linear glucose and optimized glycerol feeding strategies during induction ensured stable, scalable enzyme production.

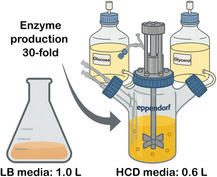

## 1 Introduction

The arylmalonate decarboxylase (AMDase) was first discovered and isolated in 1990 from the soil bacterium *Bordetella bronchiseptica* (*Bb*) during an enzymatic screening aimed at identifying catalysts capable of asymmetric decarboxylation of disubstituted malonates [1]. The *Bb*AMDase catalyzes a cofactor‐independent single‐step decarboxylation of prochiral arylmalonic acids. While the natural substrate remains unknown, the broad substrate spectrum enables the stereoselective synthesis of top‐selling NSAIDs, such as naproxen and flurbiprofen, from their corresponding arylmalonic acid precursors [2, 3]. Because the anti‐inflammatory effect is mediated by the (*S*)‐enantiomers, and all known wild‐type AMDases are strictly (*R*)‐selective, protein engineering has been employed to enhance activity and invert stereoselectivity. For instance, the (*S*)‐selective *Bb*AMDase variant ICPLLG (V43I/G74C/A125P/V156L/M159L/C188G) contains a repositioned catalytic cysteine in the active site. Further, the (*R*)‐selective *Bb*AMDase variant IPLL (V43I/A125P/V156L/M159L) exhibits a sixfold higher activity toward flurbiprofen malonate than the wildtype [3]. Additionally, the ancestral variant N131 has recently been reconstructed to provide greater stability [4].

AMDase is produced recombinantly in the Gram‐negative bacterium *Escherichia coli* (*E. coli*), which accounts for 30–40% of industrial enzyme production [5, 6]. The manufacturing process benefits from the fast replication rate and comparably cheap media, resulting in high intracellular product titers [7]. The *E. coli* strain BL21 (DE3) was selected for its widespread use in heterologous gene expression systems [8]. This strain contains a chromosomally integrated T7 RNA polymerase gene under the control of the lacUV5 promoter, enabling tightly regulated and high‐level transcription of target genes cloned downstream of a T7 promoter. The lacUV5 mutation reduces catabolite repression [9], thereby permitting efficient transcriptional induction even in the presence of rapidly metabolized carbon sources, such as glucose, or low‐cost substrates, including pretreated molasses [10, 11]. Production of the target protein is induced by the addition of lactose or a structural analog (e.g., IPTG) [12, 13]. Although downstream processing may require multiple purification steps following biomass harvest, the robustness and high transcriptional output of this expression system often outweigh host‐related limitations, provided that posttranslational modifications, such as glycosylation, are not required [14].

While *E. coli* remains a robust host for recombinant AMDase production, the cultivation method is critical for maximizing enzyme yield. Shake flask (SF) cultivations are commonly used in research laboratories due to their simplicity and minimal preparation time; however, they often yield insufficient amounts for large‐scale production of the target protein. The lack of control over critical process parameters, such as pH and dissolved oxygen (DO), increases variability, and the unfeasibility of feeding applications reduces overall yields. The use of (multi)bioreactors enables precise control of all parameters critical for the process, while recent advances in sensorics and artificial intelligence allow simultaneous prediction of results [15, 16].

**SCHEME 1:**
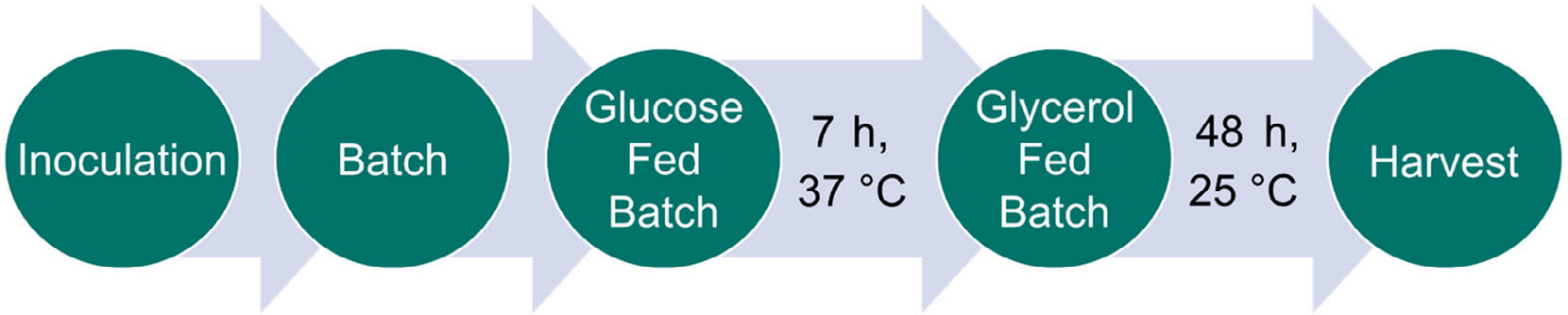
Overview of the HCDC process setup. The batch phase ended after initial glucose depletion. A linear glucose fed‐batch phase was performed for 7 h, until the glycerol fed‐batch was initiated with IPTG induction and the temperature was reduced to 25°C. After 48 h of induction, the cultivation broth was harvested. HCDC = High‐cell‐density cultivation.

To date, AMDase production has been reported exclusively in SFs using LB medium [17]. In this case study, we present the transfer of target protein expression from shake flasks to bioreactor systems, using high‐cell‐density cultivation (HCDC) to maximize yield (Scheme 1). The HCDC method was developed using the DASGIP Parallel Bioreactors (Eppendorf, Germany) to significantly enhance AMDase production. Given the system's complexity, the HCDC protocol was designed not only to achieve higher enzyme titers but also to provide a general cultivation protocol.

This study aimed to develop a Standard Operating Procedure that can be readily adapted to alternative expression systems, such as those utilizing the *araBAD* promoter or lactose induction, with minor modifications (e.g., partial substitution of glycerol with lactose or arabinose). A critical component of this strategy is the transition to glycerol as the primary carbon source during the induction phase, which helps to prevent catabolite repression in certain *E. coli* strains [18, 19]. Although glucose is the preferred carbon source for *E. coli* [20], its relatively high cost limits its industrial application. In contrast, glycerol, an inexpensive by‐product of biodiesel production [21], offers favorable biomass‐to‐substrate yields [19]. In the HCDC process, glucose is employed during the initial growth phase to support rapid biomass accumulation. Subsequently, the induction phase is decoupled from growth by switching to glycerol, a less preferred but cost‐effective and more slowly metabolized carbon source.

## 2 Results and Discussion

### 2.1 Shake Flask Cultivation

Recombinant AMDase production was performed using four distinct *E. coli* BL21 (DE3) strains to evaluate the robustness of the cultivation strategy. These included the wildtype *B. bronchiseptica* AMDase, two engineered *Bb* variants ((*S*)‐selective *Bb*‐ICPLLG and (*R*)‐selective *Bb*‐IPLL), and the ancestral variant N131 ( Table S1) [4]. First, we cultivated *E. coli* strains to produce AMDase in SFs as a benchmark for comparison with HCDCs. The current SF protocol uses LB medium (1 L medium in a 5 L cultivation flask) with IPTG induction (22 h at 28°C). The wet‐cell weight (WCW) and mass‐specific activity of the cell‐free extract (CFE) at the end of cultivation were determined for four AMDase variants (Table 1). SF cultivations yielded WCW values ranging from 6.0 g⋅L^−1^ to 7.3 g⋅L^−1^. The observed differences in mass‐specific activity of the CFE are attributable to the properties of the AMDase variants. The lowest activity was observed by the (*S*)‐selective ICPLLG variant [22], while the other (*R*)‐selective AMDase variants show increased activity by one order of magnitude.

**TABLE 1:** WCW and mass‐specific activity values obtained from SF cultivations were determined for four different AMDase variants. Reaction conditions: 100 µL CFE (1:100 diluted) in 900 µL phenylmalonic acid (22.2 mM in 50 mM Tris‐HCl, pH 8); 1300 rpm, 30°C, 4.25 min total assay time. Measured in technical triplicates. The total protein concentration of CFE was determined using the BCA assay.

AMDase	WCW, g⋅L^−1^	Specific activity, U⋅mg^−1^
wildtype^a^	6.43	146.7 ± 8.5
ICPLLG^a^	6.90	15.2 ± 5.4
IPLL^a^	6.03	134.7 ± 2.4
N131^b^	7.27	92.5 ± 2.4

a From *Bordetella bronchiseptica*.

b Ancestral sequence reconstructed AMDase [4] (Supporting Information (SI), Section 2.1).

### 2.2 High‐Cell‐Density Cultivation—Process Configuration

Next, AMDase production was performed as HCDC using the DASGIP Parallel Bioreactor system with four stirred‐tank reactors. Achieving high cell densities requires the use of bioreactors to effectively control several critical process parameters. This includes control of temperature, pH, DO, stirring speed, foam formation, as well as media composition and feeding rates. To achieve this, each bioreactor is equipped with four programmable peristaltic pumps, enabling pH control, antifoam addition, and feeding during cultivation.

HCDCs pose challenges that are not typically encountered in SF experiments. At the onset of the process, substrate inhibition caused by certain medium components can impair cell growth (Table S6). In later stages, the accumulation of metabolic by‐products, such as acetate, can further suppress cell proliferation [23, 24]. To address this, the developed (HCDC) process was conducted in a semichemically defined medium (Table S5) designed to balance high substrate availability with the risk of substrate inhibition. pH stabilization was achieved through one‐sided control using an ammonia solution, which also functioned as an auxiliary nitrogen source. This one‐sided pH regulation was deemed sufficient because acetate is the primary by‐product formed during overflow metabolism at high carbon source concentrations.

Regarding process stability, foam prevention is critical due to limitations in reactor volume and the associated risk of obstructing aeration filters. This issue becomes particularly pronounced during the later stages of cultivation, as the oxygen demand increases in high‐cell‐density cultures. Especially during the induction phase, the cultivation is prone to foaming. The amount of antifoam added to the fermentation should be limited to minimize potential adverse effects on recombinant protein production. Additionally, the oxygen mass transfer coefficient $k_{L}a$ can be reduced, necessitating higher stirring speeds and increased air supply to compensate [25]. Moreover, oxygen limitation leads to mixed‐acid fermentation in *E.coli* [26, 27], whose products must be neutralized by base addition, thereby increasing the cultivation volume. The installation of foam‐breaking impellers may help mitigate foam accumulation during HCDC. To ensure sufficient oxygen supply, the DO cascade for HCDC described by Glaser et al. [28] (Table S3) was implemented.

The HCDC was organized into a three‐stage process (Scheme 1) with the schedule detailed in (Table S5). Beginning with the inoculation of the batch phase, glucose depletion was indicated by an increase in DO after ≈6 h. The second stage involved an automated fed‐batch phase, initiated upon observation of an increase in DO. We adapted the linear, increasing feed protocol of Glaser et al. [28] to achieve high cell densities with minimal increase in cultivation volume. While the process remained stable with respect to pH control and foam formation, the initial glucose feed protocol was shortened to 7 h to align with the work schedule, while still achieving a high cell density (OD_600_ = 70–80) before induction.

The third cultivation stage was initiated with a glycerol feed, and the temperature was then reduced to 25°C to minimize inclusion body formation. DO was reduced to 30% to minimize foam formation during protein expression. The observation of diauxic carbon source consumption was provoked with the addition of 6 mL of glycerol‐rich media (250 g⋅L^−1^). Induction was performed with IPTG (1 mM), and continuous glycerol feeding was performed with a programmed feed profile (Equation (S1)). During enzyme expression, multiple additions of vitamins, trace elements, and magnesium sulfate were made manually using a syringe to prevent precipitation in the feed medium.

### 2.3 Glycerol Feeding Strategy

**FIGURE 1:**
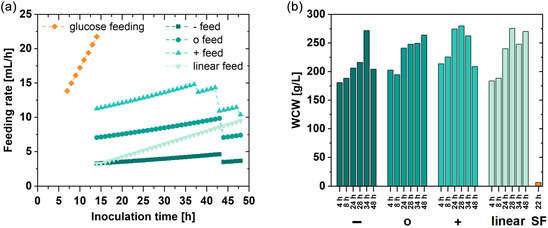
(a) Visualization of the glucose growth phase applied to all bioreactors and subsequent assessment of glycerol feeding strategies to produce *Bb*AMDase. Glucose feeding (orange) was not varied across approaches. The feeding rate was calculated hourly based on the cultivation volume (Equation (S1)). (b) WCW [g⋅L^−1^] of different feeding strategies during increased induction time, compared to the SF cultivation using LB media after 22 h induction phase*.* Feeding strategies: [‐] reduced feeding, [o] normal feeding, [+] increased feeding, [linear] linear feeding strategy, (SF) SF. SF = Shake flask.

The first experiment served to determine an optimized glycerol feeding strategy and induction time for recombinant protein production of *Bb*AMDase. Four parallel bioreactors were operated under identical conditions until the induction phase, during which four distinct glycerol feeding strategies were tested, as shown in Table 2 and in Figure 1a. The influence on the WCW of the four AMDase strains was determined, with the growth curves shown in Figure 1b.

**TABLE 2:** Glycerol feeding profiles with their respective flow rates of the glycerol‐rich feeding media (250 g⋅L^−1^) at the beginning of the induction phase until harvest. [‐], [o], and [+] were defined as static feed, accounting for the increase in volume during feeding. The increasing feed [linear] was defined as the gradient of [‐] to [+]‐feed. The flow rates [mL⋅min^−1^] are shown in Figure 1 and were calculated as described in the SI (Equation (S1)).

Feeding strategy	Description	Flow rates, start to end mL·min^−1^
[‐]	Reduced feed	3.26–4.29
[o]	Normal feed	7.05–8.69
[+]	Increased feed	11.26–12.26
[linear]	Increasing feed	3.26–12.26

Overall, the feeding strategy had only a minor impact on WCW, whereas cultivation time was the primary factor influencing biomass accumulation. The highest WCW, 279.7 g⋅L^−1^, was achieved under increased feeding conditions after 28 hours of cultivation, followed by a decline in WCW at later time points. Although the increased feed resulted in the maximum WCW, the reduced feeding strategy proved to be the most efficient in terms of resource utilization and demonstrated the highest process stability. Notably, even under reduced feeding conditions, a 43‐fold increase in WCW was observed compared to SF cultivations.

Notably, the WCW did not increase steadily over time but showed pronounced fluctuations at the end of cultivation with the reduced‐ and increased‐glycerol feeds. Since the dynamic increase of biomass was not adjusted in the feed calculation, insufficient feeding to sustain biomass after 34 h could have occurred at the reduced feeding rate. With the increased feeding rate, cell death may be associated with overfeeding of the culture, resulting in metabolic stress and increased cell death. Lysed cells are not retained in the pellet after centrifugation, which would explain the decrease in WCW. Furthermore, the broth of the HCDC becomes heterogeneous due to the high concentration of cells ( Figure S4), which could have also led to inhomogeneous sampling.

**FIGURE 2:**
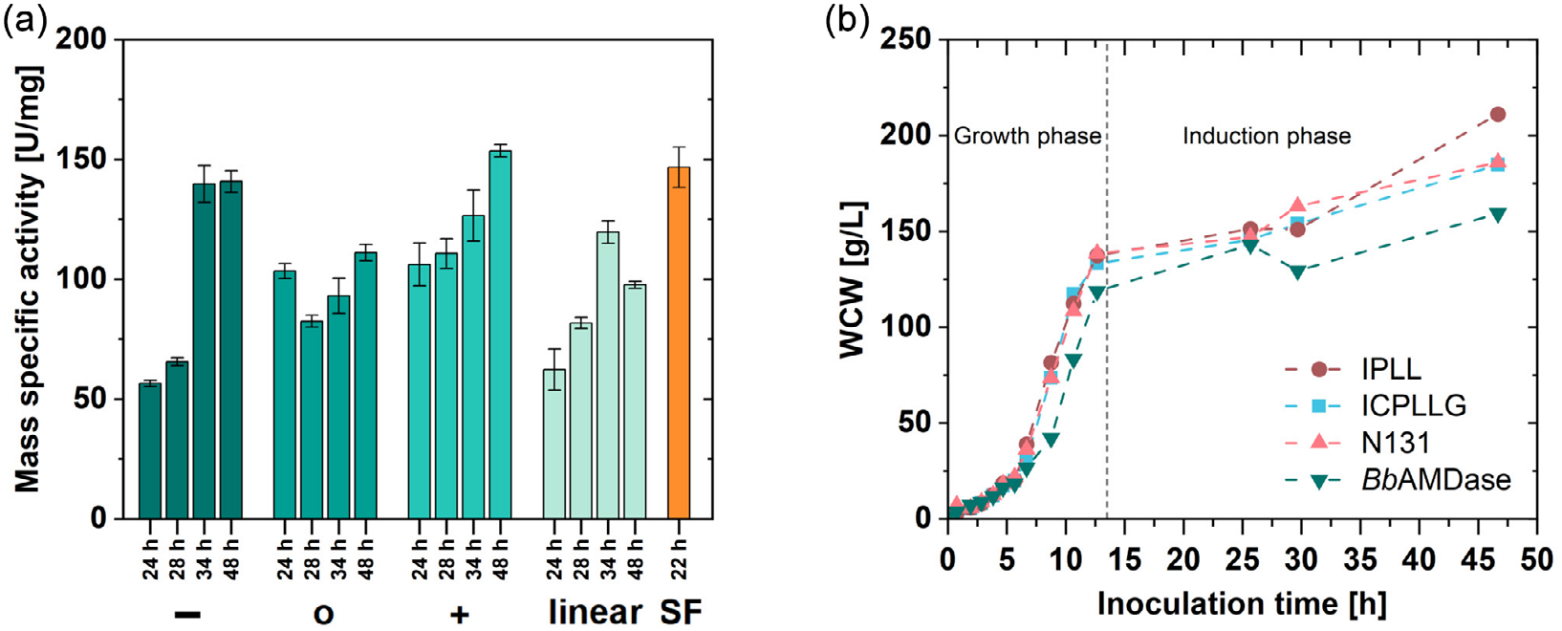
(a) Mass‐specific activity [U⋅mg_CFE_^*–*1^] of *Bb*AMDase (wildtype) detected in differing strategies over increasing induction time, compared to SF (SF) cultivation. CFE was received from 100 mg of each cell pellet. Reaction conditions: 100 µL CFE (1:100 diluted) in 900 µL phenylmalonic acid (22.2 mM in 50 mM Tris‐HCl, pH 8); 1300 rpm, 30°C, 4.25 min total assay time. Analyzed by HPLC and activity assay measured in triplicate. The total protein concentration of CFE was determined with BCA assay. Feeding strategies: [‐] reduced feeding, [o] normal feeding, [+] increased feeding, [linear] linear feeding strategy, (SF) SF. (b) Development of WCW using the reduced feeding strategy of *Bb*AMDase (wildtype), ICPLLG‐ and IPLL‐variant, and N131‐AMDase ancestor [4] during the glucose growth phase and induction phase. CFE = Cell‐free extract; SF = shake flask; WCW = wet cell weight.

In addition to biomass production, the mass‐specific activity of the CFE was evaluated and compared with that from SF cultivations. Since notable AMDase expression was achieved after 24 h of HCDC, the specific activity was analyzed at 24 h, 28 h, 34 h, and 48 h of induction time (Figure 2a). The SF cultivation achieved a mass‐specific activity of 146.7 U⋅mg^‐1^, corresponding to the total protein content of the CFE. The reduced and increased feeding strategy resulted in comparable activity with no significant difference. In contrast, the normal feeding strategy and the linear increasing glycerol feed showed similar results with lowered mass‐specific activity of 111.1 U⋅mg_CFE_^−1^ and 119.6 U⋅mg_CFE_^−1^.

Sodium dodecyl sulfate–polyacrylamide gel electrophoresis (SDS–PAGE) analysis (Figure S1) for qualitative evaluation of protein expression revealed dominant overexpression across all feeding strategies. The soluble fraction of the CFE was further analyzed by densitometric analysis of SDS–PAGE gels, with bovine serum albumin (BSA) as a calibration standard, to determine protein expression levels (Table 3). All feeding strategies resulted in an increase in soluble overexpression, reaching over 40% of total CFE protein after 28 and 34 hours with reduced feed. The highest protein expression yields were observed under an increased feeding strategy after 34 hours, with 46.6%. Despite this observed trend, the methodology is subject to a high degree of error. Small pipetting errors result in high variability on the SDS–PAGE gel. Nevertheless, these results align with literature reports for protein expression with T7 promoter expression systems, which typically achieve expression levels ranging from 10% to 50% of total protein content [29]. Furthermore, the soluble overexpression yield is comparable to that obtained in SF cultivation with a 22‐hour induction phase, at 40.3%.

**TABLE 3:** Relative enzyme expression yields of *Bb*AMDase [% of total CFE protein] with different feeding strategies determined at the progressing time of induction. The expression yield is calculated on the soluble protein determined by BCA.

	Relative *Bb*AMDase expression yield % of total CFE protein			
Feeding strategy	24 h	28 h	34 h	48 h
Reduced	27.2	32.9	41.8	36.5
Normal	33.2	37.3	36.9	36.0
Increased	28.6	42.4	46.6	42.5
Linear	26.3	41.9	23.8	36.0

Due to its superior process stability—characterized by reduced foam formation and minimal volume increase—the lower feeding rate was selected for subsequent experiments. The 34‐hour induction phase under the reduced feeding strategy yielded satisfactory expression levels and mass‐specific enzyme activity, in addition to achieving high WCW.

### 2.4 Production of Different AMDases

Since reduced feeding was identified as the best cultivation strategy, it was applied to cultivate the AMDase ancestor N131 and the IPLL‐ and ICPLLG‐AMDase variants. The mass‐specific activity of the various AMDase variants was evaluated and compared to the SF cultivations (Figure 2). All HCDC runs carried out for AMDase production showed increased biomass production compared with the respective SF cultivations, ranging from a 42‐fold increase in *Bb*AMDase to a 25‐fold increase in ancestor N131. However, AMDase ICPLLG and IPLL did not achieve activities comparable to those from their respective SF cultivations, indicating expression problems. In addition to technological irregularities, this could also result from biological variability, which has not yet been determined. Further investigation of enzyme expression was performed by SDS–PAGE analysis of the soluble and insoluble fractions (Figure S2). All cultivated strains showed an AMDase band in the insoluble protein fraction, indicating inclusion body formation, whereas the AMDase IPLL variant band was most pronounced among the four types produced. The formation of inclusion bodies indicates fast metabolism leading to incomplete folding of the target protein, or is caused by unfavorable expression conditions (e.g., temperature, pH, or cellular stress) [30]. Further studies with temperature optimization could reduce the formation of inclusion bodies.

For a direct comparison of methodology between SFs and HCDC, the total activity of the full‐cultivation biomass was calculated and normalized to the respective protein concentration of the CFE, with only active soluble protein assessed. The observed difference in protein concentration is attributable to incomplete cell disruption. This minimizes deactivation due to sonication‐induced energy input, consistent with the reported stability of *Bb*AMDase [31]. The normalized total activity received shows a 30‐fold improvement in *Bb*AMDase and a 19‐fold improvement in the AMDase ancestor N131. In contrast, the engineered mutants, such as ICPLLG and IPLL, exhibited 27‐ and 35‐fold increases in biomass but only 13‐ and 11‐fold increases in total activity (Table 4). This significant discrepancy points to compromised soluble production. While *Bb*AMDase and N131 also displayed some divergence between biomass and active enzyme levels, the effect was far less pronounced than in the *Bb* mutants. This result aligns with the stability–activity trade‐off often observed in protein engineering [32], where mutations selected for enhanced intrinsic function (f) can destabilize the protein scaffold. As previously described, this loss of stability likely reduces the fraction of protein that can fold into a soluble, functional protein (${\left[E\right]}_{0}$) [33]. As a consequence of the intensified protein production in high‐density environments, the increased load on the protein folding machinery likely amplified the accumulation of misfolded intermediates, leading to a reduced concentration of functional protein via aggregation.

**TABLE 4:** HCDC compared to SF cultivations of the same cultivation volume (1 L) of different AMDase types produced regarding their mass‐specific activity [U⋅mg^‐1^], WCW [g⋅L^−1^], and the normalized total activity [U⋅mg_Lysat_^−1^]. Reaction conditions: 100 µL CFE (1:100 diluted) in 900 µL phenylmalonic acid (22.2 mM in 50 mM Tris‐HCl, pH 8); 1300 rpm, 30°C, 4.25 min total assay time. Analyzed by HPLC. Activity assay measured in triplicates. The total protein concentration of CFE was determined with BCA. SF = shake flask, HCD = high‐cell‐density.

AMDase *Cultivation type*		Mass‐specific activity, U⋅mg^−1^	WCW, g⋅L^−1^		Normalized total activity, U⋅mg_Lysat_^−1^	Fold improvement
wildtype^a^ (*SF* )	146.7 ± 8.5		6.4	12.7 ± 0.6		29.8
wildtype^a^ (*HCD*)	139.71 ± 7.7		271.0	378.6 ± 20.7		
ICPLLG^a^ (*SF* )	15.2 ± 2.4		6.9	1.4 ± 0.1		12.9
ICPLLG^a^ (*HCD*)	9.8 ± 0.5		184.7	18.1 ± 0.9		
IPLL^a^ (*SF* )	134.7 ± 2.38		6.0	10.8 ± 0.7		11.0
IPLL^a^ (*HCD*)	56.4 ± 1.4		211.2	119.2 ± 2.8		
N131^b^ (*SF* )	92.5 ± 5.3		7.3	9.0 ± 0.4		18.5
N131^b^ (*HCD*)	89.6 ± 3.9		186.0	166.8 ± 7.3		

a from *Bordetella bronchiseptica*.

b Ancestral sequence reconstructed AMDase [4] (Supporting Information, Section 2.1).

### 2.5 Mechanistic Insights: Metabolic Burden and Carbon Flux Dynamics

To understand why this anabolic bottleneck persists during *Bb*AMDase production despite the metabolic capacity of the host, the energetic and flux dynamics of the glucose‐to‐glycerol switch must be considered. Although glycerol has a lower theoretical adenosine triphosphate (ATP) yield per mole than glucose (≈15 vs. ≈26 mol ATP⋅mol^−1^ substrate), recent studies in *E. coli* BL21 (DE3) utilizing T7‐based expression systems indicate that metabolic burden is often characterized by a persistent accumulation of ATP rather than its depletion [34]. This ATP accumulation, frequently observed alongside elevated levels of fructose‐1,6‐bisphosphate and pyruvate, suggests a metabolic mismatch where the catabolic supply of energy outpaces the capacity of the host's anabolic machinery (specifically ribosomes and chaperones) to utilize it [35]. Consequently, the energy charge (ATP/ADP ratio) during the *Bb*AMDase induction phase likely remained stable or elevated, as the producing cells entered a state comparable to a carbon overfeeding response [35]. Furthermore, the switch to glycerol induced a functional reconfiguration of the tricarboxylic acid (TCA) cycle flux. In contrast to oxidative glucose metabolism, glycerol‐grown *E. coli* exhibit a downregulation of isocitrate dehydrogenase (icd), effectively reducing carbon flux through the decarboxylation steps of the TCA cycle to minimize CO_2_ loss [36]. This shift, combined with the activation of the glyoxylate shunt in BL21, facilitates the preservation of carbon for the synthesis of essential four‐carbon precursors required to sustain the high demand for amino acid biosynthesis during recombinant expression [36]. Thus, the transition to glycerol was not merely a substrate substitution but a strategic optimization of the host's central metabolism to accommodate the high‐level production of *Bb*AMDase.

The implementation of a glycerol‐fed induction phase was specifically designed to circumvent the common bottlenecks of acetate accumulation and carbon catabolite repression. In typical glucose‐based batch cultivations of E. coli, overflow metabolism can lead to acetate concentrations exceeding 10 g/L, which severely inhibits growth and protein production [37]. In contrast, glycerol is a nonphosphotransferase system carbon source whose transport and phosphorylation are not coupled to the dephosphorylation of EIIA‐Glc [38]. This metabolic pathway ensures that intracellular cyclic adenosine monophosphate (cAMP) levels remain high during the induction phase, promoting robust transcription from the T7/lacUV5 promoter by facilitating the formation of the cAMP–cAMP receptor protein (CRP) complex [38]. While direct measurements of fermentation by‐products were not performed in this study, the efficacy of this strategy is supported by established literature for *E. coli* BL21(DE3) in HCDC. Unlike K12‐derived strains (e.g., JM109 or MG1655), *E. coli* BL21(DE3) possesses a more active glyoxylate shunt and constitutively high levels of acetyl‐CoA synthetase (ACS) during growth, which allows it to efficiently recycle acetate into the TCA cycle [39]. Literature values for similar glycerol‐fed processes indicate that acetate concentrations typically remain below 0.5–1.0 g⋅L^−1^, even at cell densities exceeding 100 g⋅L^−1^[37]. The robust biomass development attained in this study (reaching 280 g⋅L^−1^ WCW) and the stability of the pH‐stat feeding strategy further corroborate the claim that overflow metabolism and its associated catabolite repression were effectively bypassed.

## 3 Conclusion

In the present study, the production of various AMDase variants was successfully transitioned from simple SF cultivations in LB medium to a three‐stage HCDC process. Implementation of an optimized glycerol feed strategy during the induction phase resulted in a 30‐fold increase in the yield of active, soluble enzyme without increasing cultivation volume. The methodology was also successfully applied to multiple *E. coli* strains for AMDase production, consistently yielding at least a one‐order‐of‐magnitude improvement. However, the comparison of engineered variants also highlighted a critical generalizable aspect regarding protein fitness (W), defined as the product of the concentration of functional protein and its specific activity ($W={\left[E\right]}_{0}\cdot f)$ [33]. While previous studies of protein engineering have enhanced catalytic activity (f), the observed reduction in unit yield indicates a concurrent decline in the concentration of folded protein (${\left[E\right]}_{0}$). This likely occurs because less successful folding of engineered mutants limits soluble production in high‐density environments [33]. Consequently, the presented protocol serves not only as a robust production template for cofactor‐free enzymes in *E. coli*, but also as a tool to evaluate the trade‐off between intrinsic activity and process stability. In future studies, further process optimization is warranted, and adaptation to alternative expression systems warrants evaluation.

## Supporting Information

The authors have cited additional references within the Supporting Information. Additional SI can be found online in the Supporting Information section.

## Funding

This work was supported by Niedersächsisches Ministerium für Wissenschaft und Kultur (Grant AUFF).

## Conflicts of Interest

The authors declare no conflicts of interest.

## Supporting information

Supplementary Material

## Data Availability

The data that support the findings of this study are available from the corresponding author upon reasonable request.
