# Supplementary material for: High‐Yield Expression of Arylmalonate Decarboxylase in Escherichia coli Through High‐Cell‐Density Cultivation Strategies
Source: Chembiochem. 2026 Apr 21;27(8):e70339. doi: 10.1002/cbic.70339 (PMC13096762; doi:10.1002/cbic.70339)
Supplement: Supplementary file 1 — Supplementary Material [file CBIC-27-e70339-s001.pdf]

Supporting Information

High-Yield Expression of Arylmalonate Decarboxylase in *E. coli* Through High-Cell-Density Cultivation Strategies

Jan Gerstenberger,[a] Timm Werbilo,[a] Robert Kourist,[b] and Selin Kara*[a, c]

In memory of Prof. Iván Lavandera García, our beloved and dear colleague and friend.

[a] J. Gerstenberger, T. Werbilo, Prof. Dr.-Ing. habil. S. Kara
Institute of Technical Chemistry
Leibniz University Hannover
Callinstraße 5, 30167 Hannover, Germany
E-mail: selin.kara@iftc.uni-hannover.de

[b] Prof. Dr. R. Kourist
Institute of Molecular Biotechnology
Graz University of Technology
Petersgasse 14, 8010 Graz, Austria

[c] Prof. Dr.-Ing. habil. S. Kara
Biocatalysis and Bioprocessing Group
Department of Biological and Chemical Engineering
Aarhus University
Gustav Wieds Vej 10, 8000 Aarhus C, Denmark
E-mail: selin.kara@bce.au.dk

Supporting Information

TABLE OF CONTENTS

1. SDS-PAGES 3

2. METHODS 4

2.1 *ESCHERICHIA COLI* STRAINS 4
2.2 OVERNIGHT-CULTURES 4
2.3 SHAKE FLASK CULTIVATIONS 4
2.4 HIGH CELL DENSITY CULTIVATIONS 4
2.5 WET CELL WEIGHT DETERMINATION 9
2.6 CELL DISRUPTION 9
2.7 BRADFORD ASSAY 9
2.8 BCA-ASSAY 9
2.9 SDS-PAGE 9
2.10 QUALITATIVE INCLUSION BODY DETERMINATION 10
2.11 HIGH-PERFORMANCE LIQUID CHROMATOGRAPHY 10
2.12 ACTIVITY ASSAY OF CELL FREE EXTRACT 10
2.13 COMPOSITION OF MEDIA AND BUFFERS 12

3. CHEMICALS 15

4. REFERENCES CITED IN THE SUPPORTING INFORMATION 16

1. SDS-PAGE

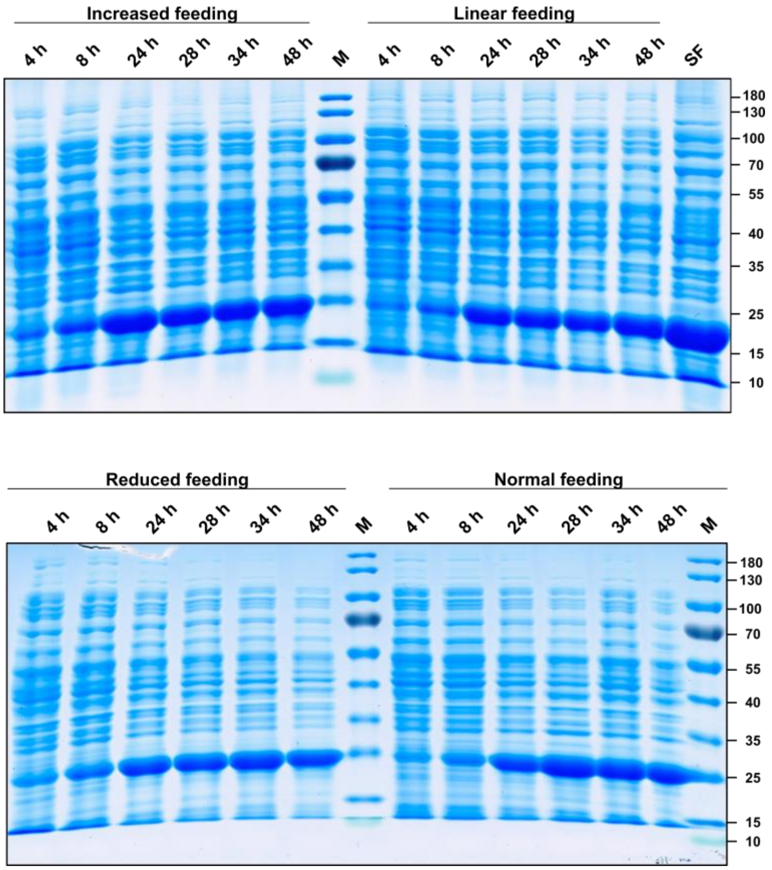

Figure S1. SDS-PAGE of CFE from all feeding strategies with increasing induction time, protein amount normalised to 10 µg. SF: Shake flask cultivation. M: Marker (10–180 kDa, Thermo Scientific, Cat. No.: 26616), *Bb*AMDase = 26 kDa.

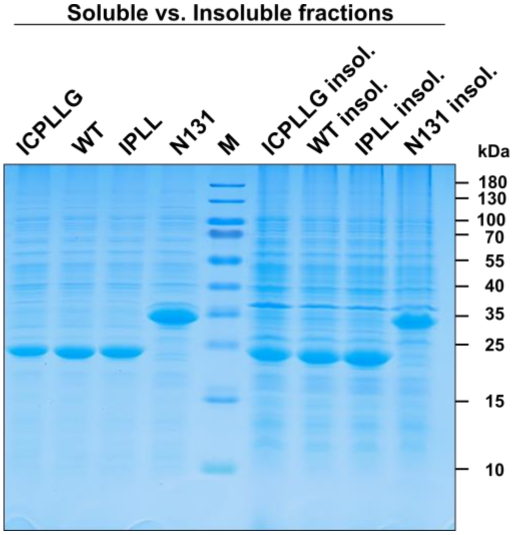

Figure S2. SDS-PAGE of soluble and insoluble fractions of four AMDase variants. ICPLLG-AMDase (24 kDa), *Bb*AMDase (24 kDa), IPLL-AMDase (24 kDa), N131-AMDase (34 kDa). The suspended sample of the insoluble fraction was normalised to the hypothetical protein concentration of the respective sample and incorporated also as a suspension in the preparation of SDS-Page. M: Marker (10–180 kDa, Thermo Scientific, Cat. No.: 26616).

2. METHODS

2.1 *Escherichia coli* strains

Recombinant protein expression of AMDase was performed in *E. coli* BL21 (DE3). The plasmid information of *Bb*AMDase, ICPLLG- and IPLL variants[40], as well as AMDase ancestor N131[4], are listed in **Table S1**.

Sequence of AMDase ancestor N131:

>N131

MTTPVIGMIVPPAAGEVPPEAYGLYPEGVRFAARGLALKELSMEDYSEAIERVAELARELREEEGADAISL
MGTSLSFFRGGAFNDELVEIMQQETGVPATTMSNSIRDALRAVGARRIAVGTAYTDEVNDRLRGFLEASG
FEVASLTGMGLTAVEDILAVTEDEVTDLGLRAFEAADGPADAVLISCGGLPALHLADALEPEIGVPVVASST
AGVWGAVRLLGLSGESPALGRLGRTPPSRESAVGHHHHHH*

Table S1. The information on plasmids and genotype of AMDase variants[40] and of AMDase N131 ancestor.[4]

Strain	Genotype
Escherichia coli BL21(DE3)	F⁻ omp T gal dcm lon hsdSB(rB--mB-)λ(DE3[lacl lacUV5-T7 gene 1 ind1 sam7 nin5])
**Plasmid**	
pET28a_*Bb*AMDase	Insert of AMDase from *Bordetella bronchiseptica*, T7 promotor, Kanamycin-Resistance, C-terminal His_6 Tag
pET28a_AMDase ICPLLG	Insert of AMDase-ICPLLG-Mutant, T7 promotor, Kanamycin-resistance, C-terminal His_6 Tag
pET28a_AMDase IPLL	Insert of AMDase-IPLL-Mutant, T7 promotor, Kanamycin-resistance, C-terminal His_6 Tag
pET28a_AMDase N131	Insert of AMDase-N131 ancestor, T7 promotor, Kanamycin-resistance, C-terminal His_6 Tag

2.2 Overnight-cultures

Overnight cultures (ONC) of *E. coli* BL21 (DE3) to produce AMDase (*Bb*, IPLL, ICPLLG, and N131) were inoculated with the addition of 20 µL glycerol stock to 15 mL lysogeny broth (LB)-media (kanamycin 40 µg/mL) and incubated (37 °C, 120 rpm, 16 h).

2.3 Shake flask cultivations

For shake flask cultivations, 25 mL of ONC was used to inoculate 1 L of LB medium (OD_{600} = 0.1; kanamycin, 40 µg/mL; 120 rpm, 37°C). Induction was started (1 mM IPTG) at an OD_{600} of 0.9. The main culture was incubated for 22 h (28 °C, 70 rpm). The cells were then harvested and centrifuged (RCF = 10000 g, 20 min, 4 °C). The cell pellet was weighed and stored at -20 °C until further use.

2.4 High cell density cultivations

High-cell-density cultivations of *E. coli* BL21 (DE3) for AMDase production were carried out in a DASGIP® Parallel Bioreactor System. Four fermenter vessels were equipped with an overhead stirrer with two Rushton impellers, a pH sensor, a DO sensor, a temperature sensor, a level sensor for antifoam addition, media feeding, and gassing, and a sample port. Unused ports were closed with a blind stopper. Cleaning, sterilization of tubes, and calibration of pumps and pH- and DO-sensors were controlled using DASware software for each bioreactor. The fermenter vessels were filled with the high-cell-density (HCD) medium

(600 mL, **Table S5**) and autoclaved separately (20 min, 121 °C). After autoclaving, the fermenter vessels were placed in the Bioblock, and the impeller motors were attached to the vessel lids. For inoculation, 25 mL of ONC of the respective strain was prepared as described and added through a septum. After depletion of glucose (approx. 7 h after inoculation), the first fed batch phase was initiated. The procedure described by Glaser *et. al*.[28] was shortened to 7 h. Feeding with glucose-rich medium was discontinued, and a change of carbon source was initiated by reducing the temperature to 25 °C and adding 6 mL of the glycerol-rich medium. The feed of glycerol-rich media was hourly pre-calculated by

$$F = \frac{\mu \cdot X \cdot V}{c_{\mathrm{Feed}} \cdot Y_{\mathrm{XS}}} \tag{S1}$$

with the assumption of a stable cell population after induction. In **Equation (S1)** where F is the feeding rate [mL/h], μ is the specific growth rate [h^{-1}] (fixed at 0.05 h^{-1}), X is the estimated biomass concentration at the start of induction [g/L], V is the reactor volume [L], c_{Feed} is the concentration of the glycerol feed [g/L] and Y_{XS} is the biomass to substrate yield [g/g] (set at 0.35 g/g for glycerol).[41] Biomass was estimated from OD_{600} values using the conversion factor $X = 0.29 \cdot \mathrm{OD}_{600}$ based on previous experiments. For practical reasons, parameters such as X, μ, and Y_{XS} were not dynamically adjusted. The feeding was started after the diauxic behaviour was observed, with simultaneous induction (1 mM IPTG). During enzyme expression, multiple additions of vitamins, trace elements, and magnesium sulphate were made manually using a syringe to prevent precipitation in the glycerol feed medium.

Due to maximal bioreactor volume limitations, 150 mL of culture was removed before induction. Additional volume reductions were performed as described in **Table S2** during the induction phase to avoid surpassing the maximum working volume. For harvesting, the cell suspension was centrifuged (RCF = 10000 g, 20 min, 4 °C). The supernatant was discarded, and the pellet resuspended in Tris-HCl-buffer (50 mM, pH 8). Afterwards, the cell suspension was aliquoted and centrifuged again (RCF = 10000 g, 20 min, 4 °C). The final cell pellets were weighed and frozen at -20 °C until further use.

Table S2. Overview of glycerol feeding strategies and reactor volume adjustments during the induction phase.

Feeding strategy	Glycerol feed rate at start – end [mL/h]	Reactor volume at induction [mL]	Volume reduction [mL] after induction [h]
**Reduced feed [-]**	3.26–4.29	609.7	150 mL at start<br>100 mL at 30 h
**Normal feed [o]**	7.05–8.69	607.5	150 mL at start<br>250 mL at 30 h
**Increased feed [+]**	11.26–12.26	609.7	150 mL at start<br>110 mL at 23 h<br>300 mL at 30 h<br>100 mL at 34 h
**Linear feed [linear]**	3.26–12.26	610.2	150 mL at start<br>250 mL at 30 h<br>100 mL at 34 h

Table S3. DO cascade for high cell density cultivation reported by Glaser *et al.*[28]

	Controller output		Actuator output	
**Agitation**	X1	0%	Y1	500 rpm
	X2	50%	Y2	1,500 rpm
**Gassing rate**	X1	50%	Y1	36 sL/h
	X2	75%	Y2	120 sL/h
**XO_2**	X1	75%	Y1	21%
	X2	100%	Y2	46%

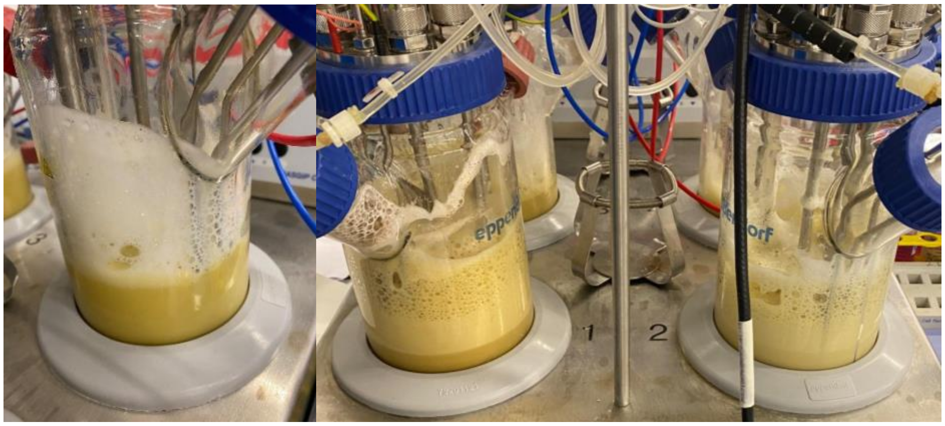

Figure S3. Excessive foam formation occurs during high-cell-density cultivation (HCDC).

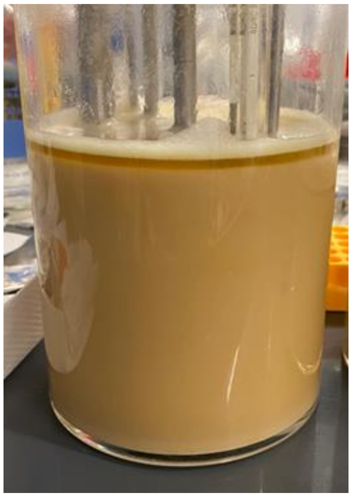

Figure S4. Sedimented cell broth of a high cell density cultivation (HCDC) at harvest.

Table S4. Schedule of high-cell-density cultivation.

**Preparation beforehand**		1. Preparation of batch and fed batch media<br>2. Assembly of the fermenter vessels<br>3. Definition of workflow in DASware control<br>4. Mounting vessel & functionality test
**Day 1**	Morning<br>11:00<br><br><br><br><br>17:00	1. pH probe calibration<br>2. Autoclaving of fermenter vessel with media inside<br>3. Pump calibration (if needed<br>4. Assembly after autoclaving<br>5. Clean in Place (CIP)<br>6. Final Assembly<br>7. Preparation of overnight cultures
**Day 2**	08:00<br><br>10:00<br>~16:00<br><br>23:00	1. Setting the fermentation parameters in software<br>2. DO probe calibration<br>3. Inoculation and start of batch phase<br>4. Observation of glucose depletion<br>5. Start of the fed-batch phase (7 hours)<br>6. Addition of Glycerol (Observation of diauxic consumption) & temperature and DO reduction<br>7. Volume reduction (150 mL)<br>8. Induction with IPTG, Start of 2nd fed batch phase (34 hours)
**Day 3**		1. Taking samples<br>2. Preparation for harvesting
**Day 4**	09:00	1. Harvesting<br>2. Clean in Place (CIP)<br>3. Autoclaving and cleaning of the fermenter vessels.

2.5 Wet cell weight determination

For the determination of wet cell weight, samples were taken from the sample port of the bioreactor, and 1 mL of the suspension was centrifuged (10000 rpm, 10 min, 4 °C). The remaining supernatant was carefully removed from the cell pellet using a pipette, and the wet cell biomass was weighed.

2.6 Cell disruption

Cell disruption was performed with sonication. The cells (100 mg/mL) were resuspended in 1 mL TRIS-HCl buffer (50 mM, pH 8, 4 °C). The sonication (amplitude 60%, pulse mode: 2 s pulse, 8 s pause, 2 min) was carried out on ice, and the disrupted cells were centrifuged (13000 rpm, 40 min, 4 °C). The supernatant was collected and used directly for further experiments.

2.7 Bradford Assay

The Bradford assay was performed in a 96-well plate. 20 µL of the sample was mixed and incubated with 200 µL of Bradford reagent (15 min, 22 °C). End-point measurements were performed in a plate reader, using the 595 nm and 470 nm absorption wavelengths for linearization. Samples were applied in duplicate, and protein content was quantified using a calibration curve with BSA (0–2 mg/mL).

2.8 BCA-Assay

The BCA assay was performed using Pierce™ BCA Protein Assay Kit and carried out in 96-well plates. 200 µL of Working Reagent (Reagent A 50:1 Reagent B) was mixed in a well with 25 µL of the sample and incubated (30 min, 37 °C). End-point measurement was performed using a plate reader at 562 nm. Samples were applied in duplicate, and protein content was quantified using a calibration curve with BSA (0–2 mg/mL).

2.9 SDS-PAGE

Gel electrophoresis was done in Bio-Rad electrophoresis chambers with 12% acrylamide gels. The composition of the separating and stacking gel is shown in **Table S13**.

The prepared gels were clamped into Bio-Rad frames, and the chamber was filled with TGS buffer. The samples were mixed with Laemmli buffer (3:1) and incubated (500 rpm, 6 min, 95 °C). The wells were loaded with 5–8 µL of sample, depending on the protein concentration of the normalised samples. 3 µL of pre-stained protein ladder (10–180 kDa, Thermo Scientific, Cat. No.: 26616) was used. Electrophoresis was performed at 90 V for 15 min, followed by a voltage increase to 120 V. The gel was washed with ddH_2O, and stained with Coomassie Blue for 16 h. Subsequently, the gel was de-stained with ddH_2O, after which it was scanned and pre-processed.

For densitometric SDS-PAGE, the protein content of all samples was normalised, and protein concentration was subsequently analysed using the BCA assay. The samples and a calibration curve with BSA (55.5–222.2 µg/mL) were applied to the gel at 6 µL. After scanning, protein content was analyzed using Gel-Analyzer 19.1 software (**Figure S5**).

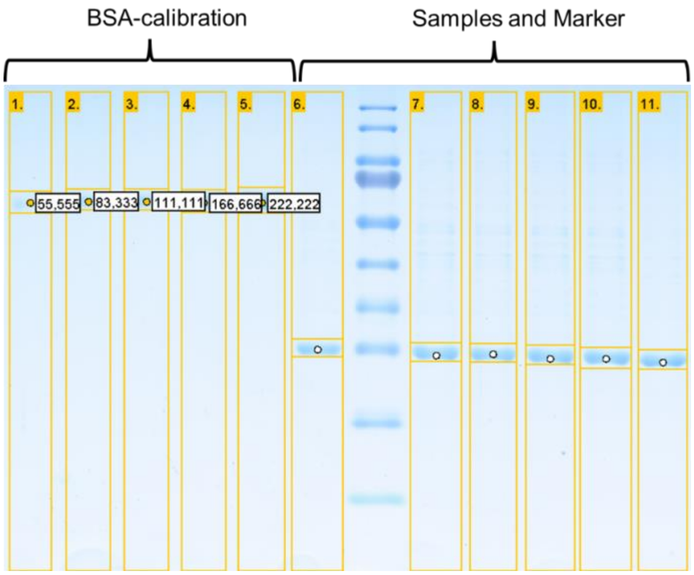

Figure S5. Exemplary densitometric analysis of protein content in Gel Analyzer software.

2.10 Qualitative inclusion body determination

After sonication, the lysed cells were centrifuged (1 mL sample, 13000 rpm, 4 °C, 20 min), and the protein concentration of the CFE was determined using the BCA assay. The samples were diluted for SDS-PAGE analysis to load 10 µg of total protein per pocket. The remaining pellets of inclusion bodies and cell debris were washed five times with ddH_2O (1 mL, 13000 rpm, 4 °C, 15 min). The suspended sample was normalised to the hypothetical protein concentration of the respective sample and also incorporated as a suspension in the preparation of SDS-PAGE.

2.11 High-performance liquid chromatography

HPLC measurements were performed using a VWR® Hitachi Chromaster equipped with a 5110 pump, 5210 autosampler, 5310 column oven, and 5430 diode-array detector. To quantify product concentration, high-performance liquid chromatography (HPLC) was performed. A Kinetex® 2.6 µm Polar C18 100 Å column with an inner diameter of 4.6 mm and a length of 150 mm was used. The samples were measured with a flow rate of 1 mL/min using a gradient of H_3PO_4 (20 mM) in ddH_2O and acetonitrile (95:5 → 20:80%, 15 min). The non-linear calibration of phenylacetic acid (25 µM–10 mM, pH 8) was prepared in TRIS-HCl buffer (50 mM) to determine product formation using HPLC with absorbance at 210 nm.

2.12 Activity Assay of Cell Free Extract

The determination of enzyme activity was carried out in 1.5 mL reaction tubes (30 °C, 700 rpm). For this, substrate stock solution (22.22 mM, TRIS-HCl buffer 50 mM, pH 8) was mixed 1:10 with diluted CFE (TRIS-HCl buffer 50 mM, pH 8, t_{start} of assay. The reaction tubes were incubated while shaking (30 °C, 700 rpm) and samples (100 µL) were collected at 00:15 , 01:15 , 02:15 , 03:15 , and 04:15 min. The reaction was terminated by addition to a mixture of 200 µL ACN:ddH_2O (9:1). The samples were centrifuged (4 °C, 10000 rpm, 20 min). and analysed with HPLC. For the determination of the activity of the sample, the rise of product formation was plotted against time, and the slope was determined (**Figure S6**).

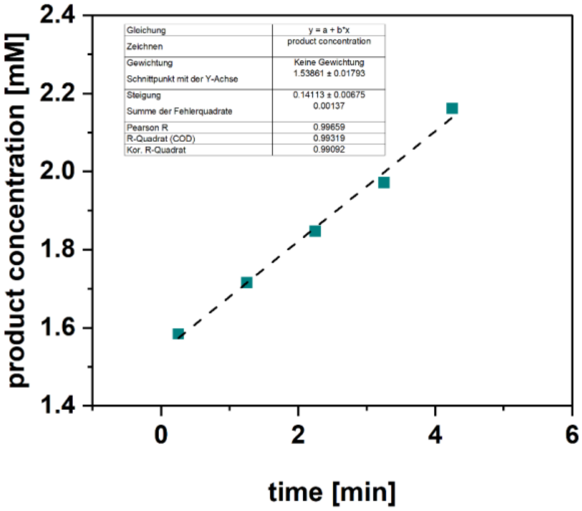

Figure S6. Determination of slope through linear regression.

For the determination of mass-specific activity $a_{\mathrm{spec.}}$ [U/g$_{\mathrm{CFE}}$], the total protein concentration in the sample was calculated by the total protein determined by the BCA-assay of the CFE. The activity of the sample a_{sample} [U] was divided with the used total protein content m_{CFE} [mg$_{\mathrm{CFE}}$] in the sample as seen in **Equation (S2)**.

$$a_{\mathrm{spec.}} = \frac{a_{\mathrm{sample}}}{m_{\mathrm{CFE}}} \tag{S2}$$

The total activity a_{total} [U/L] was calculated by the activity of the sample a_{sample} [U] multiplied with the volume of CFE V_{CFE} receivable from the whole cultivation biomass regarding the described protocol above.

$$a_{\mathrm{total}} = a_{\mathrm{sample}} \cdot V_{\mathrm{CFE}} \tag{S3}$$

The normalised total activity $a_{\mathrm{normalised\ total}}$ [U/mg$_{\mathrm{Lysat}}$] was determined by dividing the total activity by the protein concentration c_{CFE} [g$_{\mathrm{Lysat}}$/L] of the respective CFE.

$$a_{\mathrm{normalised\ total}} = \frac{a_{\mathrm{total}}}{c_{\mathrm{CFE}}} \tag{S4}$$

2.13 Composition of media and buffers

Table S5. Composition of high cell density batch medium and glucose feed media. The components of the HCD stock solution and the glucose stock solution were dissolved in ddH_2O at 50 °C and filtered through a 0.22 µm filter. The $MgSO_4$ stock solution was autoclaved. Vitamin and trace element stocks are self-sterile due to their pH. During glycerol feeding (250 g/L glycerol, 40 µg/mL kanamycin), micronutrients ($MgSO_4$, Vitamin stock solution, and trace elements) were manually supplemented to the bioreactor at the same composition as the glucose-rich feed medium to prevent precipitation in the feeding lines. The volume was calculated to match the final concentrations shown below, based on the flow rate determined using **Equation (S1)**. The final concentrations are calculated on the total volume of the specific media (finished batch media or the glucose feed, which is added), with their respective component concentrations (g/L).

Agent		Stock-solution	Batch		Glucose Feed	
		**Mass [g]**	**Final concentration (g/L)**	**Amount (mL)**	**Final concentration (g/L)**	**Amount (mL)**
**ddH_2O**		-	-	524.4	-	-
**HCD stock (10x)**	NH_4Cl	2	0.2	60.0	0.2	20.0
	$(NH_4)_2SO_4$	20	2.0		2.0	
	KH_2PO_4	130	13.0		13.0	
	K_2HPO_4	100	10.0		10.0	
	$NaH_2PO_4 \cdot H_2O$	52.2	6.0		6.0	
	Yeast extract	30	3.0		3.0	
	ddH_2O	*Ad* 1 L	-		-	
**$MgSO_4 \cdot 7\ H_2O$ (400 g/L)**		400	1.0	1.5	10.0	5.0
**Glucose · H_2O (600 g/L)**		600	8.0	8.0	518.4	172.8
**Vitamin stock (40 mL)**	Biotin	0.0001	$5.0 \cdot 10^{-6}$	3.0	$5.0 \cdot 10^{-6}$	1.0
	Folic acid	0.0002	$1.0 \cdot 10^{-5}$		$1.0 \cdot 10^{-5}$	
	Pyridoxin-HCl	0.05	$2.5 \cdot 10^{-3}$		$2.5 \cdot 10^{-3}$	
	Thiamine-HCl	1	$5.0 \cdot 10^{-2}$		$5.0 \cdot 10^{-2}$	
	Riboflavin	1	$5.0 \cdot 10^{-2}$		$5.0 \cdot 10^{-2}$	
	Nicotinic acid	0.05	$2.5 \cdot 10^{-3}$		$2.5 \cdot 10^{-3}$	
	Calcium D-pantothenate	0.05	$2.5 \cdot 10^{-3}$		$2.5 \cdot 10^{-3}$	
	Vitamin B12	0.001	$5.0 \cdot 10^{-5}$		$5.0 \cdot 10^{-5}$	
	HCl (5 M)	*Ad* 100 mL	-		-	
**Trace elements stock (40 mL)**	$MnSO_4 \cdot H_2O$	0.1625	$1.3 \cdot 10^{-2}$	2.4	$1.3 \cdot 10^{-2}$	0.8
	AlCl3	0.0897	$1.3 \cdot 10^{-2}$		$1.3 \cdot 10^{-2}$	
	$FeSO_4 \cdot 7H_2O$	0.625	$5.0 \cdot 10^{-2}$		$5.0 \cdot 10^{-2}$	
	$CoCl_2 \cdot 6H_2O$	0.11	$8.8 \cdot 10^{-3}$		$8.8 \cdot 10^{-3}$	
	$CaCl_2 \cdot 2H_2O$	0.472	$5.0 \cdot 10^{-2}$		$5.0 \cdot 10^{-2}$	
	$ZnSO_4 \cdot 7H_2O$	0.0313	$2.5 \cdot 10^{-3}$		$2.5 \cdot 10^{-3}$	
	$CuCl_2 \cdot 2H_2O$	0.0129	$1.3 \cdot 10^{-3}$		$1.3 \cdot 10^{-3}$	
	H_3BO_3	0.0079	$6.3 \cdot 10^{-4}$		$6.3 \cdot 10^{-4}$	
	$Na_2MoO_4 \cdot 2H_2O$	0.0313	$2.5 \cdot 10^{-3}$		$2.5 \cdot 10^{-3}$	
	NaOH (1 M)	*Ad* 50 mL	-		-	
**Antifoam solution (20%)**			0.17	0.1	-	-
**Kanamycin (40 mg/mL)**			0.05	0.6	0.05	0.2
**Total**			-	600.0	-	200.0

Table S6. Inhibitory concentrations of important macronutrients and trace elements in batch media.[23, 42]

Substrate	Inhibitory concentration [g/L]
Glucose	50
Ammonia	3
Phosphorus	10
Magnesium	8.7
Molybdenum	0.8
Boron	0.044
Copper	0.0042
Manganese	0.068
Cobalt	0.059
Zinc	0.038
Iron	1.15

Table S7. Kanamycin antibiotics stock solution. The 1000x stock was filtered through a 0.22 µM filter and stored at -20 °C.

Agent	Amount
**Kanamycin**	400 mg
**ddH_2O**	10 mL

Table S8. Composition of glycerol feed media. The solution was filtered to 0.22 µM and then filtered into a 2 L feed bottle prior to cultivation.

Agent	Amount [g] or [mL]
**Glycerol**	500 g
**HCD Stock**	4 mL
**Kanamycin stock solution (40 mg/mL)**	2 mL
**ddH_2O**	*ad* 2000 mL

Table S9. IPTG stock solution for protein expression. The 1000x stock was filtered through a 0.22 µM filter and stored at -20 °C.

Agent	Amount
**IPTG**	2.3818 g
**ddH_2O**	10 mL

Table S10. Antifoam emulsion. The components were added to a 250 mL feed bottle together with a stir bar and autoclaved. The feed bottle was stirred at 400 rpm throughout cultivation to prevent phase separation.

Agent	Amount
**Antifoam KS 911**	50 mL
**ddH_2O**	200 mL

Table S11. 50 mM TRIS-HCl buffer composition. The buffer was stored at 4 °C until usage.

Agent	Amount
**TRIS-HCl**	6.06 g
**conc. HCl**	2.35 mL
**pH**	adjust to 8
**ddH$_2$O**	*ad* 1000 mL

Table S12. HPLC eluent composition. The described gradient (**section 3.7**) was set as the method in the HPLC. Acetonitrile was used as delivered (VWR International, USA).

Agent	Amount
**H$_3$PO$_4$ (85%)**	6.74 mL
**ddH$_2$O**	*ad* 5000 mL
**Ultrasound sonication**	20 min

Table S13. Preparation of Stacking and Separation gel (12% acrylamide). The finished gels were stored at 4 °C for up to 3 weeks.

Gel	Agent	Amount [mL]
**Separation gel**	**ddH$_2$O**	6.21
	ROTIPHORESE®Gel 30	13.5
	TRIS-HCl (1.5M, pH 8.8)	6.75
	SDS 10%	0.27
	APS 10%	0.27
	TEMED	12 µL
**Stacking gel**	**ddH$_2$O**	8.10
	PAGE 30%	2.01
	TRIS-HCl (1.5M, pH 6.8)	1.50
	SDS 10%	0.12
	APS 10%	0.12
	TEMED	12 µL

3. CHEMICALS

Table S14. Chemicals used in this case study.

Chemical	Supplier
Acetonitrile	VWR International, USA
Albumin Standard Ampules	Thermo Fisher Scientific Inc., USA
Aluminium trichloride	Carl Roth GmbH & Co. KG, Germany
Ammonium sulphate	Carl Roth GmbH & Co. KG, Germany
Antifoam KS 911	Evonik Tego Chemie GmbH
APS	Carl Roth GmbH & Co. KG, Germany
BCA reagents	Thermo Fisher Scientific Inc., USA
Biotin	Sigma-Aldrich, USA
Boric acid	Fluka Chemika, Switzerland
Calcium chloride hexahydrate	AppliChem GmbH, Germany
Calcium D-panthothenate	Carl Roth GmbH & Co. KG, Germany
Cobalt(II) chloride hexahydrate	Carl Roth GmbH & Co. KG, Germany
Coomassie-Brilliant Blue G250	SERVA Electrophoresis GmbH, Germany
Copper(II) chloride dihydrate	Carl Roth GmbH & Co. KG, Germany
Dipotassium hydrogen phosphate	VWR International, USA
Ethanol	Carl Roth GmbH & Co. KG, Germany
Folic acid	Fluka Chemika, Switzerland
Glucose	Merck KGaA, Germany
Imidazole	AppliChem GmbH, Germany
Iron(II) sulphate heptahydrate	Carl Roth GmbH & Co. KG, Germany
Isopropyl-β-D-Thiogalaktopyranosid	AppliChem GmbH, Germany
Kanamycin sulfate	Amresco, USA
Magnesium sulfate heptahydrate	Carl Roth GmbH & Co. KG, Germany
Manganese sulphate monohydrate	Carl Roth GmbH & Co. KG, Germany
Nicotinic acid	Carl Roth GmbH & Co. KG, Germany
pH buffer 4, 7 and 9	Carl Roth GmbH & Co. KG, Germany
Phenylmalonic acid	Tokyo Chemical Industry Co., Ltd., Japan
Phosphoric acid (85%)	Carl Roth GmbH & Co. KG, Germany
Potassium dihydrogen phosphate	VWR International, USA
Pyridoxin-HCl	Carl Roth GmbH & Co. KG, Germany
Riboflavin	Fluka Chemika, Switzerland
Sodium dodecyl sulfate (SDS)	Carl Roth GmbH & Co. KG, Germany
Sodium dihydrogen phosphate monohydrate	Carl Roth GmbH & Co. KG, Germany
Sodium hydroxide	VWR International, USA
Sodium molybdate dihydrate	Carl Roth GmbH & Co. KG, Germany
Thiamine-HCl	Carl Roth GmbH & Co. KG, Germany
TRIS-HCl	Carl Roth GmbH & Co. KG, Germany
Vitamin B12	Carl Roth GmbH & Co. KG, Germany
Yeast extract	Thermo Fisher Scientific Inc., USA
Zinc sulphate heptahydrate	Carl Roth GmbH & Co. KG, Germany

4. REFERENCES CITED IN THE SUPPORTING INFORMATION

[4] E. van der Pol, J. Gerstenberger, X. Georgiadou, K. Schliep, C. Schür, S. Kara, R. Kourist, “Ancestors of Arylmalonate Decarboxylase Show Increased Activity, Stability and Stereoselectivity” *bioRxiv preprint* **2026**, DOI: 10.64898/2026.01.14.699310.

[23] D. Riesenberg, “High-Cell-Density Cultivation of Escherichia Coli” *Curr. Opin. Biotechnol*. **1991**, *2*, 380–384.

[28] R. Glaser, A. Niehus, M. Sha, “High-Density Escherichia Coli Fed-Batch Fermentation Using the SciVario® Twin Bioreactor Control System”, Application Note, Eppendorf, Hamburg (DE), **2022**.

[40] M. Aßmann, PhD Thesis, Technische Universität Hamburg (DE), **2019**.

[41] R. Román, N. Lončar, A. Casablancas, M. W. Fraaije, G. Gonzalez, “Optimized production of active fungal peroxygenase in *E. coli* inclusion bodies” *Appl. Microbiol. Biotechnol.* **2020**, *104*, 5337–5345.

[42] T. Majtan, F. E. Frerman, J. P. Kraus, “Effect of temperature on the folding and stability of cystathionine β-synthase: mechanism of pyridoxal 5'-phosphate's effect” *BioMetals* **2011**, *24*, 335–347.
